# Measurement of Electrical Properties of Superconducting YBCO Thin Films in the VHF Range

**DOI:** 10.3390/ma14123360

**Published:** 2021-06-17

**Authors:** Yakir Dahan, Eldad Holdengreber, Elichai Glassner, Oz Sorkin, Shmuel E. Schacham, Eliyahu Farber

**Affiliations:** 1 Department of Electrical and Electronic Engineering, Ariel University, Ariel 40700, Israel; yakir533@gmail.com (Y.D.); elichaig@gmail.com (E.G.); ozsor@ariel.ac.il (O.S.); schacham@ariel.ac.il (S.E.S.); e.farber@ariel.ac.il (E.F.); 2 Department of Mechanical Engineering and Mechatronics, Ariel University, Ariel 40700, Israel; 3 Department of Physics, Ariel University, Ariel 40700, Israel

**Keywords:** HTSC, YBCO, VHF, microstrip resonator, quality factor

## Abstract

A new measurement technique of electrical parameters of superconducting thin films at the Very High Frequency (VHF) range is described, based on resonators with microstrip (MS) structures. The design of an optimal resonator was achieved, based on a thorough theoretical analysis, which is required for derivation of the exact configuration of the MS. A theoretical model is presented, from which an expression for the attenuation of a MS line can be derived. Accordingly, simulations were performed, and an optimal resonator for the VHF range was designed and implemented. Production constraints of YBa_2_Cu_3_O_7_ (YBCO) limited the diameter of the sapphire substrate to 3″. Therefore, a meander configuration was formed to fit the long λ/4 MS line on the wafer. By measuring the complex input reflection coefficients of a λ/4 resonator, we extracted the quality factor, which is mainly affected by the dielectric and conductor attenuations. The experimental results are well fitted by the theoretical model. The dielectric attenuation was calculated using the quasi-static analysis of the MS line. An identical copper resonator was produced and measured to compare the properties of the YBCO resonator in reference to the copper one. A quality factor of ~$6\cdot 10^{5}$ was calculated for the YBCO resonator, three orders of magnitude larger than that of the copper resonator. The attenuation per unit length of the YBCO layer was smaller by more than five orders of magnitude than that of the copper.

## 1. Introduction

Superconductors are taking an increasing role in electrical circuitry [1,2,3]. Due to their very low attenuation, numerous new measurement techniques have been introduced to characterize these materials [4,5,6,7,8]. Attenuation is a primary material parameter in electronics. It is of particular interest in circuitry implemented in novel materials such as high critical temperature superconductors (HTSC) [9,10].

By measuring the input impedance of a resonator, the attenuation can be derived [11]. In addition, it is possible to obtain a second fundamental parameter: the quality factor, Q, which is proportional to the ratio between the energy stored in the circuit to its power loss. Q is a measure of the loss, and the lower the loss, the higher the quality factor [11,12,13].

Numerous studies report on applications of superconducting resonators, either as filters, modulators, or as a vehicle for material characterization [14,15,16,17,18,19,20,21,22,23,24,25,26]. Electrical parameters, such as input impedance, attenuation, and quality factor, were derived using a Transmission Line (TL) resonator, implemented as quarter-wavelength (λ/4) Micro Strip (MS) line resonator, and loaded by a short circuit. These applications cover a wide frequency spectrum, in the ~1–50 GHz range. We introduce a new approach for performing microwave measurements for characterizing superconducting layers at the VHF range. We adopted the MS line resonator as a tool for deriving the electrical material parameters. However, since we are dealing with a much lower frequency range, the VHF, meaning a much longer wavelength range, the resonator has to be redesigned. A theoretical investigation, starting with electromagnetic analysis, is presented in Section 2, from which we derived the expressions for the input impedance and attenuation. Accordingly, we built the model for the simulations. Based on the results of these extensive simulations, an optimal design of a resonator for the VHF range was defined, as presented in Section 3. Using the simulation results, a YBa_2_Cu_3_O_7_ (YBCO) MS line resonator was implemented, on which the measurements were performed, as described in Section 4. The attenuation per unit length, as well as other electromagnetic parameters of the superconductor, were extracted from the measured input impedance. For comparison purposes, we implemented and measured a copper resonator with similar geometry.

## 2. Theory

We developed an expression for the attenuation per unit length of a TL line as a function of its physical dimensions and electrical characteristics. A quarter-wavelength TL is considered as a resonator if it is loaded with a short circuit. The input impedance of a lossy TL, loaded with an impedance *Z_L_*, can be expressed as [11,12]

(1) $$Z_{in}=Z_{0}\frac{Z_{L}+iZ_{0}\tanh\left(\gamma l\right)}{Z_{0}+iZ_{L}\tanh\left(\gamma l\right)}$$

where *Z_0_* is the characteristic impedance, and *l* is the line length. The complex propagation constant is *γ = α + iβ*, *α* being the attenuation constant due to losses (*α_d_* for the dielectric and *α_c_* for the conductor), and *β* being the phase constant, or wavenumber. The input impedance of a quarter wavelength TL is given by

(2) $$Z_{in}=\frac{Z_{0}}{\alpha l+i\pi \Delta f/2f_{0}}$$

where Δ*f* is the bandwidth of the resonator, the full width at half maximum, FWHM, and *f_0_* is the resonance frequency. Measurement of the complex input reflection coefficient, Γ_in_, allows for the extraction of the input impedance using Equation (3),

(3) $$Z_{in}=Z_{0}\frac{\Gamma_{in}+1}{\Gamma_{in}-1},$$

which, in turn, allows the calculation of the quality factor of the resonator [12]. The quality factor of the resonator is defined as the resonance frequency times the ratio between the stored energy in the circuit and the power loss. The unloaded *Q* of a λ/4 resonator is given by [11]

(4) $$Q=\frac{\pi }{4\alpha l}$$

**Figure 1:**
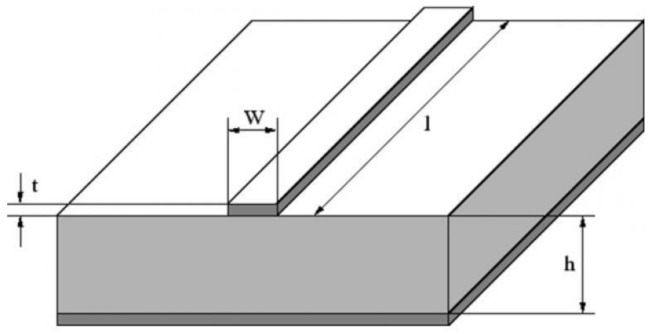
MS line geometry. The strip width is *W*, its thickness is *t*, and its length is *l*. *h* is the dielectric substrate thickness.

To evaluate the conductor losses, the dielectric losses must be calculated first. The relative dielectric constant is ε_r_ = ε_r_′ + j ε_r_′′. A MS line, shown in Figure 1, consists of a conducting strip separated from a conducting ground plane by a dielectric layer which serves as the substrate for the strip. *W* is the strip width, *l* is its length, *t* is its thickness, and the thickness of the dielectric is *h*. TL is considered as “inhomogeneous” since the strip is not surrounded by the dielectric material. This inhomogeneity can be taken into account by introducing an effective dielectric constant, ε_e_, given by

(5) $$\varepsilon_{e}=\frac{\varepsilon_{r}+1}{2}+\frac{\varepsilon_{r}-1}{2}{\left(1+\frac{12h}{W}\right)}^{-\frac{1}{2}}+F\left(\varepsilon_{r},h\right)-0.217\left(\varepsilon_{r}-1\right)\frac{t}{\sqrt{Wh}},$$

where:

(6) $$F\left(\varepsilon_{r},h\right)=\left\{\begin{array}{c} 0.02\left(\varepsilon_{r}-1\right){\left(1-\frac{W}{h}\right)}^{2},\;\;\frac{W}{h}<1\\ 0,\;\;\frac{W}{h}>1 \end{array}\right.,$$

Accordingly, the dielectric losses can be derived from

(7) $$\alpha_{d}=\frac{\pi }{\lambda_{0}}\frac{\varepsilon_{r}}{\sqrt{\varepsilon_{e}}}\frac{\varepsilon_{e}-1}{\varepsilon_{r}-1}\tan\left(\delta \right),$$

where λ_0_ is the vacuum wavelength of the radiation at the resonance frequency, and tan (δ)$=\frac{\varepsilon_{r}’}{\varepsilon_{r}’’}$ are the dielectric losses, given by 2·10^−7^ in the sapphire substrate [27]. Using Equations (5) and (6), we derived an effective dielectric constant of ε_e_ = 6.076, while the quasi-static numerical solution of Kirschning and Jansen [28] renders ε_e_ = 6.044, a difference of less than 1%.

## 3. Simulations and Design

Characterization of attenuation at the VHF frequency range, using a quarter-wavelength MS line resonator, imposes a practical challenge due to the very long wavelength. At the frequency of 50 MHz, around which we performed our measurements, λ/4 ~ 50 cm. The implementation of the MS line with HTSC, such as YBCO, is challenging since the production of YBCO layers are usually limited to a diameter of about 3″ [29]. In order to fit the MS line to the wafer, a meander-line was carefully implemented [30,31].

By preforming over 100 simulations, the optimal design of the MS resonator, with the desired frequency response, was obtained. The goal of the simulations was to minimize the radiation loss of the MS line and the coupling effect between the adjacent MS meander lines. The electromagnetic simulations were performed using Dassault Systèmes CST Studio Suite software, while the analysis of the input impedance was preformed using Keysight Advanced Design Software (ADS). The CST is a general-purpose electromagnetic simulator, based on the Finite Integration Technique (FIT) [32]. The FIT discretizes the integral form of the Maxwell equations using the Perfect Boundary Approximation (PBA) technique [33]. Time domain simulations were done by applying a wide-band pulse to the resonator structure, and extracting the complex scattering parameters with a frequency separation of 90 kHz. The resonator structure was divided into its different components—namely, the MS line, the dielectric substrate, and the conducting box, in order to introduce them properly in the electromagnetic simulation “mesh”. For the MS line and the substrate, a hexagonal mesh with a maximum mesh size of λ/10 was selected. This mesh size was set in order to properly handle the curvature of the meander and its effect on the resonator parameters. A hexagonal mesh was used for the resonator box as well, but with a much larger mesh size of λ/4, since the box has a minimal effect on the resonator parameters. The simulations provided the optimal values for the dielectric thickness *h* = 2.82 mm, the width of the strip *W* = 0.33 mm, and the length of the strip *l* = 511 mm. The thickness of the conducting strip *t* was set by the manufacturer to either 330 nm for the superconductor or 1 μm for the metal. The physical parameters of the resonator are summarized in Table 1. For electromagnetic simulations of the superconductor, using the CST software, we used the Perfect Electric Conductor (PEC) option. For comparison purposes we performed simulations of a copper resonator of a similar structure.

**Figure 2:**
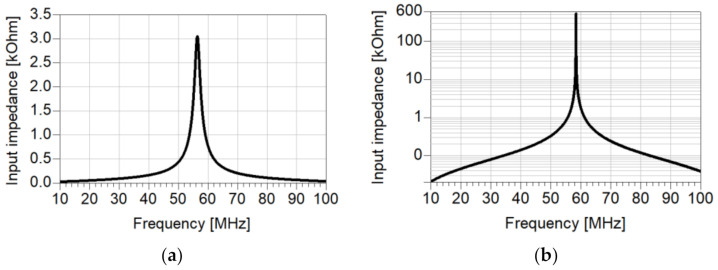
Simulated input impedance of copper (**a**, linear scale), and PEC λ/4 (**b**, logarithmic scale) resonators as a function of frequency.

For electromagnetic simulations of the superconductor using the CST software, we applied the Perfect Electric Conductor (PEC) option. For comparison, we performed simulations of a copper resonator of a similar structure [34]. The input impedances for the copper (a, in linear scale) and PEC (b, in logarithmic scale) resonators with a short circuit load are displayed in Figure 2. The obtained maximum input impedance is 3 kΩ for the copper and 530 kΩ for PEC structures. The resonance frequencies *f*_0_ of the copper and PEC resonators are 56.5 and 58.4 MHz, with FWHM Δ*f* of 1875 and 10.7 kHz, respectively. Using an equivalent definition of *Q* for a resonator [11],

(8) $$Q=\frac{f_{0}}{\Delta f}$$

We derived a quality factor of approximately 30 for the copper resonator and 5457 for the PEC one.

## 4. Measurements and Results

The electrical parameters were derived from measurements of the input impedance of the MS resonators. Both YBCO and copper resonators were implemented on sapphire substrates. Due to the production limitations on the dimensions of YBCO layers, a 3″ diameter sapphire substrate was used. We used a meander configuration to implement the long quarter-wavelength MS line dictated by the low VHF range. Since the losses of the YBCO are extremely low, it was essential to assure very low losses of the dielectric substrate [35,36]. In order to achieve a uniform critical current density, the surface of the sapphire dielectric substrate was polished to a roughness of 0.5 nm, and R-plane cut with a tolerance of ±2° and flatness of 4–5 wave per inch. As a result, the minimal critical current density was 3.23 M A/cm^2^. This result should be compared to a minimal critical current density of −1 M A/cm^2^ and a larger non-uniformity of the critical current density with a roughness of 2 nm [37]. The strips had to be passivated against oxidation. Gold coating is frequently used to protect the superconducting layer [38]. However, electromagnetic interaction between the gold and the propagating microwaves may cause losses [39,40,41]. To prevent these losses, we covered the superconducting layer with a 500 nm layer of SiO_2_ [42].

**Figure 3:**
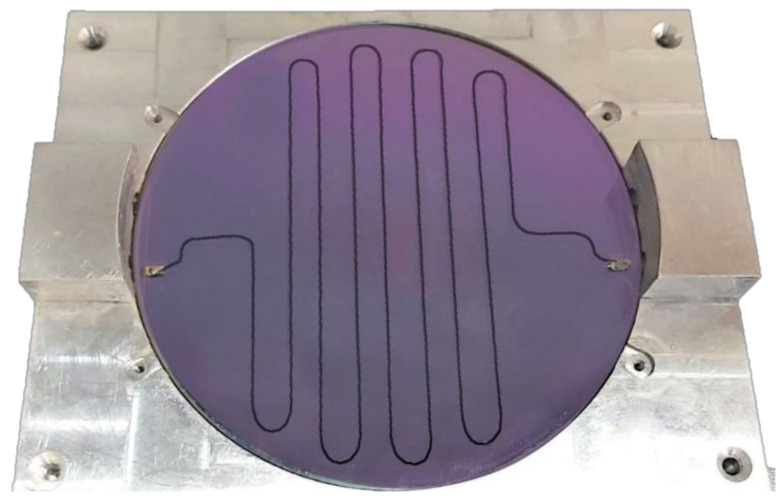
YBCO λ/4 MS meander line resonator in an aluminum housing.

The fabrication of both resonators was done by Theva GmbH [43] on the 3″ M-type sapphire wafers. For the superconducting resonator, the sapphire substrate was coated on both sides with a 330 nm YBCO film. The top side was passivated with a protective layer of 500 nm SiO_2_. A high-resolution Cr mask was used for the lithography, executed by wet etch. The copper resonator was implemented similarly, with a 1μm thick copper layer. In Figure 3, the fabricated YBCO resonator is shown in an aluminum housing. The role of the housing is twofold: to ease the attachment of the coaxial connectors and to protect the fragile circuit.

The reflection coefficient was measured using a vector network analyzer (VNA), intended for measuring the complex reflection coefficients of microwave networks. From the complex input reflection coefficient, Γ_in_, the input impedance is derived using Equation (3), which, in turn, allows the calculation of the quality factor of the resonator.

**Figure 4:**
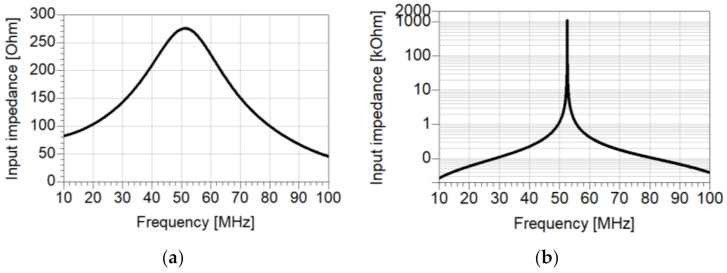
Measured magnitude of input impedance of copper (**a**, linear scale), and PEC λ/4 (**b**, logarithmic scale) resonators as function of frequency at 77 K.

The magnitude of the input impedance of the copper (a, linear scale) and HTSC resonators (b, logarithmic scale) as a function of frequency is given in Figure 4. Both measurements were performed in liquid nitrogen at 77 K. The measured maximum input impedance is 275 Ω and 1.1 MΩ, at 51.4 MHz and 52.6 MHz, with a bandwidth Δ*f* of 26.05 MHz and 9.9 kHz, for the copper resonator and for the YBCO resonator, respectively. The derived quality factor Q for the copper resonator is 1.973, and the YBCO resonator is 5342. The quality factor of the HTSC structure is more than three orders of magnitude larger.

From Equation (4), we derived the total loss of the copper resonator α^(Cu)^ = 0.671 Np/m mainly due to the conductor loss. Subtracting the dielectric loss from the total loss results in a conductor loss of α_c_^(Cu)^ = 0.67 Np/m. This loss is higher than that reported in literature [44,45,46]. However, the reported value considers the full effect of the skin depth of copper at that temperature and frequency, i.e., δ = √(2/ωµσ) = 3.06 µm. Due to the low thickness of the copper layer, which is approximately 1/3 of the skin depth, the skin effect is minimal. We calculated the total loss of the line directly from the MS line physical and electrical parameters, using Equations (9) and (10):

(9) $$\alpha =\frac{R_{s}}{tZ_{0}},$$

(10) $$R_{s}=\frac{1}{\sigma }\sqrt{\frac{\omega \mu \sigma }{2}},$$

At 77 K, the conductivity of copper is σ = 5.26·10^8^ 1/Ωm [47], while the characteristic impedance of the TL, derived from the simulations, is Z_0_ = 103.6 Ω. Taking the conductor thickness into account, we calculated the conductor loss to be 0.2 Np/m, closer to the measured attenuation. Due to the small thickness of the conductor, surface defects may cause a significant decrease in performances.

The total loss of the superconducting resonator is α_YBCO_ = 2.5515·10^−4^ Np/m as calculated using Equation (4). The main contribution to the attenuation is the dielectric loss. Using Equation (7), the conductor loss is derived to be α_c_^(YBCO)^ = 2.3533·10^−6^ Np/m, 5 orders of magnitude smaller than that of the copper resonator. The quality factor is Q = 5.8·10^5^.

The surface resistance of a superconductor, *R_s_*, has a *f^2^* frequency dependence. Based on the Mazierska’s investigation of surface resistance [45], using *R_s_* of 400 µΩ at a frequency of 10 GHz and temperature of 77 K, we estimate the surface resistance of YBCO at 50 MHz as R_s_ = 0.01 µΩ, rendering a conductor loss 0.3·10^−6^ Np/m. This result is in good agreement with the measured one.

## 5. Conclusions

A new technique for characterizing the electrical parameters of HTSC material in the VHF range is introduced. For this characterization, we adapted the configuration of a resonator with an MS structure, known to be used for resonators in the GHz ranges, for our much lower frequencies. The design of an optimal resonator was achieved, based on a thorough theoretical analysis, required for derivation of the exact configuration of the MS, starting with electromagnetic analysis, followed by extensive computer simulations.

According to the simulation results, a VHF resonator was implemented with a YBCO MS meander. The meander was required due to the very long resonance wavelength. Following the measurements performed on this high-quality resonator, we were able to accurately derive the parameters of the YBCO layer. Based on the measured input reflection coefficients, the input impedance of the resonator was derived, and the quality factor was extracted. The quality factor is mainly affected by the dielectric and conductor losses. Therefore, the dielectric loss of the MS line was calculated using the quasi-static analysis. By subtracting the effect of the dielectric losses, a quality factor of ~6·10^5^ was obtained for the YBCO resonator, three orders of magnitude larger than that of the copper resonator. The loss per unit length of the superconducting layer was smaller than that of the copper by more than five orders of magnitude. We have shown that a frequency square dependence of the surface resistivity of the superconductor is a good approximation.

## Figures and Tables

**Table 1:** Geometric parameters of the resonator.

Parameter	Value
h	2.82 mm
W	0.33 mm
t (YBCO)	330 nm
t (Copper)	1 μm
l	511 mm

## Data Availability

The data presented in this study are available on request from the corresponding author.
